# Population-Based Study of *Streptococcus suis* Infection in Humans in Phayao Province in Northern Thailand

**DOI:** 10.1371/journal.pone.0031265

**Published:** 2012-02-21

**Authors:** Dan Takeuchi, Anusak Kerdsin, Anupong Pienpringam, Phacharaphan Loetthong, Sutit Samerchea, Pakkinee Luangsuk, Kasean Khamisara, Nithita Wongwan, Prasanee Areeratana, Piphat Chiranairadul, Suwat Lertchayanti, Sininat Petcharat, Amara Yowang, Phanupong Chaiwongsaen, Tatsuya Nakayama, Yukihiro Akeda, Shigeyuki Hamada, Pathom Sawanpanyalert, Surang Dejsirilert, Kazunori Oishi

**Affiliations:** 1 Laboratory for Clinical Research on Infectious Diseases, International Research Center for Infectious Diseases, Research Institute for Microbial Diseases, Osaka University, Osaka, Japan; 2 National Institute of Health, Department of Medical Sciences, Ministry of Public Health, Nonthaburi, Thailand; 3 Chiang Kham General Hospital, Phayao, Thailand; 4 Phayao Provincial Hospital, Phayao, Thailand; 5 Phayao Public Health Office, Phayao, Thailand; 6 Chiang Rai Regional Medical Sciences Center, Chiang Rai, Thailand; 7 Thailand-Japan Research Collaboration Center for Emerging and Re-emerging Infections, Nonthaburi, Thailand; University of Iowa, United States of America

## Abstract

**Background:**

*Streptococcus suis* infection in humans has received increasing worldwide recognition.

**Methods and Findings:**

A prospective study of *S. suis* infection in humans was conducted in Phayao Province in northern Thailand to determine the incidence and the risk behaviors of the disease in this region in 2010. Thirty-one cases were confirmed. The case fatality rate was 16.1%, and the estimated incidence rate was 6.2 per 100,000 in the general population. The peak incidence occurred in May. The median age of the patients was 53 years and 64.5% were men. Consumption of raw pork products was confirmed in 22 cases and the median incubation period (range) was 2 days (0–11) after consumption of raw pork products. Isolates from 31 patients were confirmed as serotype 2 in 23 patients (74.2%) and serotype 14 in eight patients (25.8%). The major sequence types (STs) were ST1 (n = 20) for serotype 2 and ST105 (n = 8) for serotype 14. The epidemiological analysis suggested three possible clusters, which included 17 cases. In the largest possible cluster of 10 cases in Chiang Kham and its neighboring districts in May, the source of infection in four cases was identified as a raw pork dish served at the same restaurant in this district. Microbiological analysis confirmed that three of four cases associated with consumption of raw pork at this restaurant were attributable to an identical strain of serotype 2 with ST1 and pulsotype A2.

**Conclusions:**

Our data suggest a high incidence rate of *S. suis* infection in the general population in Phayao Province in 2010 and confirm a cluster of three cases in 31 human cases. Food safety control should be strengthened especially for raw pork products in northern Thailand.

## Introduction

*Streptococcus suis* is a zoonotic pathogen that can cause invasive infection in humans who have close contact with infected pigs or contaminated pork-derived products. The numbers of reported human cases, especially in Southeast Asian countries, have increased dramatically in the past few years [1]–[3]. Although serotype 2 is the most prevalent in humans, human cases involving serotypes 1, 4, 14 and 16 have been reported [1]–[5]. In a retrospective study in 2006–2008 in Thailand, *S. suis* infection was confirmed in bacterial cultures of blood or cerebrospinal fluid (CSF) from 179 patients. These isolates were determined to be serotype 2 for 165 cases (92.2%), serotype 14 for 12 cases (6.7%), and one case each (0.6%) of serotypes 5 and 24 [4]–[6]. Human infection with serotype 2 was sporadic, with a case fatality rate of 9.5% in adults, and most of these cases were located primarily in northern Thailand [4].

**Figure 1:**
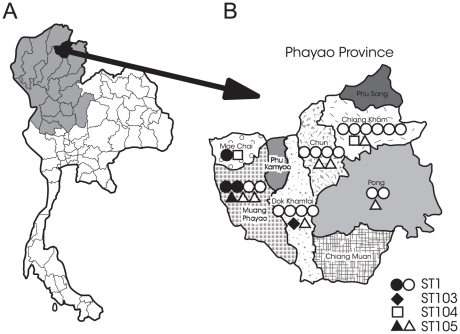
Location of the study site and distribution of human isolates. (**A**) Location of Phayao Province in northern Thailand. (**B**) Distribution and sequence typing of 31 human isolates of *Streptococcus suis* in Phayao Province in 2010 (B). One symbol is one case. Closed symbols denote fatal cases, and open symbols denote nonfatal cases.

The population of the 17 provinces in northern Thailand was 11,788,684 in 2010 (Figure 1A) [7]. Some local residents have a traditional custom of consuming raw pork dishes such as “Loo” (raw pork meat and blood), “Lap” (raw pork meat), and fermented raw pork in this region. An outbreak of *S. suis* infection including 29 laboratory-confirmed cases occurred in the Phu Sang district, Phayao Province, in northern Thailand in April and May of 2007 (Figure 1B) [8] A major route of transmission during this outbreak was the consumption of raw pig blood. This province is located close to the border with the Lao People's Democratic Republic, and the population of this province was 486,304 in 2010 [7]. Although previous studies reported that human cases of *S. suis* infection are associated with the recent consumption of raw pork products in northern Thailand and Vietnam [2]–[6], [8]–[11], the annual incidence rate of this disease in this region remains unknown.

In this study, we conducted a population-based study of *S. suis* infection in humans to determine the incidence rate of this disease in Phayao Province in 2010. We also investigated the risk behaviors of this disease and the possible clustering of cases in relation to the risk behaviors.

## Methods

### Human cases

We organized a network for the surveillance of *S. suis* infection in humans that includes the Phayao Public Health Office and two tertiary hospitals (Phayao Provincial Hospital and Chiang Kham General Hospital), and five district hospitals (Mae Chai Hospital, Chiang Muan Hospital, Dok Khamtai Hospital, Chun Hospital, and Pong Hospital); the districts within this province are shown in Figure 1B. We enrolled hospitalized patients with sepsis or bacterial meningitis when a biochemical test suggested the presence of *S. suis* in isolates from blood or CSF, and prospectively investigated the clinical and epidemiological features of the enrolled cases at these seven hospitals between January to December of 2010.

The clinical information of the enrolled case was recorded by attending physicians at a hospital in a network for the surveillance of *S. suis* infection in Phayao Province. The clinical information included the date of onset of illness and the hospital admission, and the risk behaviors, such as occupational exposures, the recent contact with pigs or raw pork products and the recent consumption of raw pork products. For the cases with the recent contact with pigs or raw pork products, the date and the location of exposure were recorded. For the cases with the recent consumption of raw pork products, the date and place of consumption of raw pork products and the type of dishes containing raw pork products were recorded. The clinical categories included meningitis and nonmeningitis based on the definition previously described [4]. The meningitis category involved confirmed meningitis, bacteremic meningitis, and probable meningitis. All patients in the meningitis category had typical meningeal signs, such as neck stiffness and an acute onset. Bacteremic meningitis was defined as a positive result in both the CSF and blood cultures, confirmed meningitis was defined as a positive culture in the CSF only, and probable meningitis was defined as a positive blood culture. The nonmeningitis category included the clinical manifestations of sepsis and sepsis with focal signs other than meningitis (septic arthritis or bacteremic pneumonia). Sepsis was defined as systemic inflammatory response syndrome with a positive blood culture.

The possible clustered cases were defined as human cases of laboratory-confirmed *S. suis* infection in combination with the recent close contact with pigs or raw pork products or with the recent consumption of raw pork products in the same or neighboring districts within 14 days of each onset of illness. This incubation period was based on a previous report of a human *S. suis* outbreak in Sichuan, China, which showed that the interval between exposure and onset is 1–14 days [12]. This population-based study of *S. suis* infection in humans was reviewed and approved by the Ethics Committees of the Department of Medical Sciences, Ministry of Public Health, Nonthaburi, Thailand. This study was conducted according to the principles expressed in the Declaration of Helsinki. The patient or guardian provided written informed consent for all cases. This study was registered at the UMIN Clinical Trial Registry (UMIN000006449).

### Microbiological study

The isolates were subjected to the following biochemical tests: API Strep (bioMérieux, Durham, NC, USA) and *S. suis*-specific and *S. suis* serotype 2- or 1/2–specific polymerase chain reaction [4], [13]. The final serotype of all strains was confirmed by coagglutination tests using rabbit antisera (Statens Serum Institute, Copenhagen, Denmark).

Multilocus sequence typing (MLST) was performed as described by King et al. [14], with a modification for *mutS* as described by Rehm et al. [15]. MLST alleles and the resulting sequence type (ST) were assigned using the *S. suis* MLST database, which can be accessed at http://ssuis.mlst.net. Pulsed-field gel electrophoresis (PFGE) was performed as described previously [16]. The pulsotypes were designated as previously described [4], and assigned to clusters of isolates with >80% similarity within the dendrogram.

### Statistical analysis

The clinical characteristics including male sex, age, risk factor, the days from the consumption of raw pork products to the onset of illness, the days from the onset of illness to the admission between fatal and nonfatal cases were compared using Fisher's exact test or Mann–Whitney *U* test with SPSS version 15.0 software. Data were considered significant for *p* values<0.05.

## Results

### Patients

**Figure 2:**
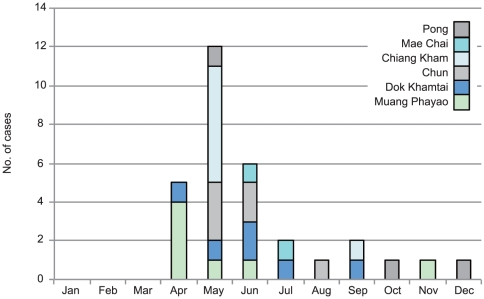
Monthly distribution of human cases of *Streptococcus suis* infection in each district in Phayao Province in 2010.

The locations of nine districts in Phayao Province and the distribution of the 31 cases in these districts are shown in Figure 1B. No case was found in the districts of Phu Sang, Phu Kamyoo, and Chiang Muan. Fatal cases were found in the districts of Muang Phayao, Dok Khantai, and Mae Chai. There was no relationship between geographical distribution of cases and the location of fatal cases. The monthly incidence of the 31 cases in each district is shown in Figure 2. The peak incidence occurred in May, and 23 cases (71.9%) were detected between April and June.

The clinical features of the 31 patients admitted with *S. suis* infection in Phayao Province between January and December 2010 are shown in Table 1. The median age (range) of these patients was 53 years (26–74) of which 64.5% were men and 35.5% were women. Five of the 31 cases (16.1%) were fatal. Recent exposure to pigs or raw pork products was noted in two cases (6.5%). One case occurred in a pork meat seller (Case 21 in Table 2) and was associated with the daily occupational exposure to the raw pork products at the wet market. Another case (Case 27 in Table 2) had the daily exposure to pigs bred at home.

**Table 1:** Clinical characteristics of 31 human cases of *Streptococcus suis* infection in Phayao Province, 2010.

Characteristics	All reported cases	Nonfatal case, n = 26; 83.9%	Fatal case, n = 5; 16.1%	*p*-value
Demographic				
Male, no. of cases (%), n = 31	20 (64.5)	18 (69.2)	2 (40)	0.317
Age, median (range), n = 31	53 (26–74)	52 (26–74)	64 (36–72)	0.115
Risk behavior, no. of cases (%)				
Recent consumption of raw pork products, n = 31	22 (71.0)	20 (76.9)	2 (40)	0.131
Recent contact with pigs or raw pork products, n = 31	2 (6.5)	2 (7.7)	0 (0)	1
Days from the consumption of raw pork products to the onset of illness median (range), n = 22	2 (0–11)	2 (0–11)*	1.5 (1–2)**	0.623
Days from the onset of illness to the admission median (range), n = 31	2 (0–14)	2 (0–7)	4 (0–14)	0.176

*n = 20,

**n = 2.

**Table 2:** Clinical, epidemiological and microbiological features of 31 human cases of *Streptococcus suis* infection in Phayao Province, 2010.

No.	Age	Sex	District	Contact with pigs or raw pork products	Consumption of raw pork products				Day of onset	Outcome	Serotype	Pulsotype*	MLST		Possible cluster
					Consumption	Date	Place	Type of product					ST complex	ST	
1	41	M	Muang Phayao	No	Yes	2 Apr	Home	Loo	5 Apr	Alive	2	A3	1	1	PC I
2	46	M	Muang Phayao	No	Yes	15 Apr	Restaurant A	Loo	17 Apr	Alive	2	A	1	1	PC I
3	72	M	Muang Phayao	No	Yes	20 Apr	Restaurant B	Loo	22 Apr	Dead	2	A	1	1	PC I
4	63	F	Muang Phayao	No	No	-			28 Apr	Dead	14	J	1	105	
5	51	M	Muang Phayao	No	No	-			15 May	Alive	14	J	1	105	
6	52	M	Dok Khamtai	No	No	-			24 Apr	Alive	14	J	1	105	
7	50	M	Chiang Kham	No	Yes	30 Apr	Home	Boiled intestine	1 May	Alive	14	J	1	105	PC II
8	56	M	Chiang Kham	No	Yes	8 May	Restaurant C	Loo	10 May	Alive	2	A	1	1	PC II
9	65	M	Chiang Kham	No	Yes	12 May	Restaurant C	Loo	13 May	Alive	2	A2^**^	1	1	PC II
10	49	M	Chiang Kham	No	Yes	12 May	Restaurant C	Loo	16 May	Alive	2	A2^**^	1	1	PC II
11	26	F	Chiang Kham	No	Yes	15 May	Restaurant C	Loo	17 May	Alive	2	A2^**^	1	1	PC II
12	53	M	Chiang Kham	No	Yes	12 May	Home	Lap	14 May	Alive	2	A2	1	1	PC II
13	59	M	Chun	No	Yes	6 May	Home	Lap	7 May	Alive	2	A2	1	1	PC II
14	57	M	Chun	No	Yes	12 May	Home	Fermented raw pork	14 May	Alive	14	J	1	105	PC II
15	54	M	Chun	No	Yes	10 May	Home	Fermented raw pork	14 May	Alive	14	J	1	105	PC II
16	45	F	Pong	No	Yes	3 May	Home	Fermented raw pork	10 May	Alive	14	J	1	105	PC II
17	72	F	Dok Khamtai	No	No	-			10 Jun	Alive	2	A1	1	1	
18	42	F	Dok Khamtai	No	Yes	26 May	Home	Loo	28 May	Alive	2	A1	1	1	PC III
19	41	M	Dok Khamtai	No	Yes	1 Jun	Restaurant D	Loo	3 Jun	Alive	2	A1	1	1	PC III
20	50	M	Chun	No	Yes	1 Jun	Home	Loo	12 Jun	Alive	2	A2	1	1	PC III
21	60	F	Chun	Yes	No	-			19 Jun	Alive	2	A2	1	1	PC III
22	52	F	Muang Phayao	No	No	-			15 Jun	Alive	14	J	1	105	
23	74	F	Mae Chai	No	No	-			20 Jun	Alive	2	H	225	104	
24	69	F	Mae Chai	No	No				20 Jul	Dead	2	A	1	1	
25	66	M	Dok Khamtai	No	Yes	2 Jul	Home	Loo	4 Jul	Alive	2	A5	1	1	
26	55	M	Chun	No	Yes	30 Jul	Home	Lap	1 Aug	Alive	2	A2	1	1	
27	34	M	Chiang Kham	Yes	Yes	30 Aug	Home	Loo	1 Sep	Alive	2	H	225	104	
28	36	M	Dok Khamtai	No	Yes	11 Sep	Home	Loo	12 Sep	Dead	2	K	29	103	
29	47	F	Pong	No	Yes	20 Oct	Restaurant E	Loo	21 Oct	Alive	2	A	1	1	
30	64	F	Muang Phayao	No	No	-			23 Nov	Dead	2	A1	1	1	
31	59	M	Pong	No	Yes	5 Dec	Home	Lap	6 Dec	Alive	2	A2	1	1	

Three possible clusters in the district of Muang Phayao (PC I), the districts of Chiang Kham, Chun and Pong (PC II), and the districts of Dok Khantai and Chun (PC III) are shown.

*The pulsotype was designated as previously described [4]. A2^**^, Serotype 2 with pulsotype A2 was the causative pathogen of a cluster of three cases. MLST, multilocus sequence type; ST, sequence typing.

Because 30 cases were confirmed in the general population of Phayao Province in 2010, the incidence rate of this disease in humans was 6.2 per 100,000 (30 in 486,304) of the general population in this province in 2010. Before the onset of illness, a recent history of consumption of raw pork products was confirmed for 22 (71.0%) of the 31 cases. A pig breeder (Case 27) also had a recent history of raw pork consumption. No information was available about the recent consumption of raw pork products or exposure to pigs or raw pork products in the remaining eight cases.

The clinical manifestations in the 31 patients included fever (n = 27; 87.1%), headache (n = 19; 61.3%), hearing loss (n = 12; 38.7%), altered consciousness (n = 9; 29.0%), and diarrhea (n = 6; 19.4%). The comorbid illnesses of these patients included alcoholic liver cirrhosis (n = 4; 12.9%), hypertension (n = 3, 9.7%), diabetes mellitus (n = 1; 3.2%), rheumatoid arthritis (n = 1; 3.2%), aplastic anemia (n = 1; 3.2%), and spinal canal stenosis (n = 1; 3.2%). No comorbid illness was found in 20 patients (64.5%). None of the demographic features, including the risk behavior of recent consumption of raw pork products and recent exposure to pigs or raw pork products, was significantly associated with a fatal outcome (Table 1). The median interval (range) between the consumption and onset of illness was 2 days (0–11) for 22 patients. The median period (range) from the onset of illness to admission was 2 days (0–14) for 31 patients. The interval from the onset of illness to admission was not associated significantly with a fatal outcome, although the interval tended to be longer in the fatal cases than in the nonfatal cases. The meningitis category (n = 20; 64.5%) included five cases of confirmed meningitis, nine cases of bacteremic meningitis, and six cases of probable meningitis. The nonmeningitis category (n = 11; 35.5%) included five cases of septic arthritis and six cases of sepsis.

### Clustered cases

We next examined whether the clustered cases that were linked epidemiologically and caused by an identical strain, were included in the 31 cases. The clinical, epidemiological and microbiological features of 31 human cases of *S. suis* infection is shown in Table 2. In 22 patients with a recent history of consumption of raw pork products, these products were consumed at home by 14 patients and at 5 different restaurants by eight patients. The most frequent dish (14/22 cases; 63.6%) was “Loo”. Three possible clusters including 17 cases associated with recent consumption of raw pork products or the recent exposure to pigs or raw pork products were found based on the case definition in the 31 cases. A possible cluster in the Muang Phayao district found in April included three cases (shown as PC I). Another possible large cluster including 10 cases was found in the districts of Chiang Kham, Chun, and Pong in May (shown as PC II). The other possible cluster including four cases was found in the districts of Dok Khamtai and Chun between May and June (shown as PC III). Interestingly, four patients visited restaurant C and consumed “Loo” in Chiang Kham district between May 8 and 15, 2010. These four patients had febrile illness 1–4 days after consuming “Loo” at this restaurant. By contrast, no epidemiological linkage was found in the other 13 cases in three possible clusters.

### Isolates of *S. suis*

*S. suis* was isolated from all 31 patients. Of the 31 isolates, 23 (74.2%) were serotype 2 and the other eight (25.8%) were serotype 14 (Table 2). The sequence typing of serotype 2 isolates were ST1 for 20 isolates (64.5%), ST104 for two isolates (6.5%), and ST103 for one isolate (3.2%). All eight serotype 14 isolates were ST105 (25.8%). In four patients in the possible large cluster (PC II in Table 2) in Chiang Kham and its neighboring district with a history of visiting restaurant C, serotype 2 strain with ST1 and pulsotype A2 was isolated from three cases, and serotype 2 strain with ST1 and pulsotype A was isolated from one case.

## Discussion

In this study, we confirmed 31 human cases of *S. suis* infection with a case fatality rate of 16.1% in Phayao Province in 2010. This case fatality rate is equivalent to that recorded previously in Thailand [4], [11], [12]. To exclude the possibility that human cases of *S. suis* infection in residents of Phayao Province were detected in hospitals in the surrounding three provinces of Chiang Rai, Lampang, and Nan, we investigated all human cases in these provinces through the hospital network surveillance system for *S. suis* infection organized by the Thai NIH in 2010 [17]. Because no human cases from Phayao Province were found in these three provinces in this surveillance, our data represent a population-based study of *S. suis* infection in humans in this province.

The incidence rate (6.2 per 100,000) in the general population in Phayao Province in 2010 is 69 times higher than that (0.09 per 100,000) in Hong Kong [18], which is the sole available data for the general population in Southeast Asian countries. By contrast, the incidence rate of this disease is as low as 0.002/100,000 in the general population in a developed country such as The Netherlands [19]. Our present data suggest that the highest incidence rate of this disease among adults in the general population in this region is associated with the habitual behavior of consuming raw pork products. Given the incidence rate of this disease and the population in northern Thailand, the estimated number of human cases can be calculated as 730 per year in this region.

The disease incidence peaked during the rainy season (June to August) in a retrospective study between 2006 and 2008 in all 76 provinces of Thailand [4]. By contrast, the peak incidence was May 2010 in our current study. A previous outbreak in the Phu Sang district, Phayao Province, was also found during April and May in 2007 [8]. The shift of the peak incidence to April and May might be related to the Songkran Festival (a traditional new year festival in Thailand) in April and other harvesting festivals during this period in this region.

A recent case–control study in southern Vietnam reported that eating undercooked pig blood or intestine within 2 weeks of the appearance of infection was the most important risk factor [20]. In our study, we also confirmed that more than 70% of cases with *S. suis* infections were associated with the recent consumption of raw pork products. Importantly, the estimated incubation period for this disease after oral consumption of raw pork products was only 2 days. This finding strongly suggests that the oral consumption of raw pork products is the major transmission route. A previous study of an *S. suis* outbreak in Sichuan, China, similarly reported a median interval of 2.2 days between exposure and onset of infection, although the transmission route in this outbreak was direct contact with the blood or tissues of sick or dead pigs [12]. No information was available about the recent history of the consumption of raw pork products or exposure to pigs or raw pork products in eight cases (25.8%), which included 6 housewives who might have been exposed unintentionally to the contaminated pork products during cooking.

Of the 31 cases in our study, 23 (74.2%) were caused by serotype 2 and eight (25.8%) were caused by serotype 14. In the previous retrospective study between 2006 and 2008, serotype 14 was confirmed in 12 of 179 human cases (6.7%) of *S. suis* infection in Thailand [5], [6]. Of these 12 cases, only one case with serotype 14 was found in Phayao Province in 2007. Our present data suggest that the prevalence of serotype 14 as a cause of human disease has increased in this province since 2009.

Molecular analyses using MLST and PFGE provided evidences of an outbreak of *S. suis* serotype 2 in Sichuan, China [21] and the clonal disseminations of *S. suis* serotypes 2 and 14 among sporadic human cases [4], [5]. In the present study, we used MLST and PFGE to investigate whether the 23 human cases found in April to June 2010 contained the clustered cases that were linked epidemiologically through the consumption of raw pork products and caused by an identical pathogen. An obvious spatial and temporal clustering of four cases was found in Chiang Kham district. All four patients (Cases 8–11) consumed “Loo” at restaurant C within the same week in May 2010. The bacteriological analysis confirmed that the four cases were caused by the isolates of serotype 2 with ST1. Of these four isolates, the pulsotypes were A2 for three cases and A for one case. Because the strain with pulsotype A was found to have <80% similarity to strain with pulsotype A2 in the dendrogram [4], this strain with pulsotype A was interpreted to be distinct from pulsotype A2. Our data suggest that the “Loo” at restaurant C were contaminated with two distinct strains of serotype 2 with ST1 and pulsotype A for Case 8 and of serotype 2 with ST1 and pulsotype A2 for Cases 9–11. A cluster comprising three cases caused by an identical serotype 2 strain with ST1 and pulsotype A2 (shown as A2^**^ in Table 2) was confirmed in Chiang Kham district in May, 2010.

Our present data also suggest that the raw pork products consumed by most of our patients were contaminated with *S. suis*. In Phayao Province, most raw pork products are supplied from the local slaughterhouses to the wet markets. A recent study of *S. suis* serotype 2 infection reported that slaughterhouse pigs were the source of infection by *S. suis* serotype 2 in southern Vietnam [22]. A previous study in Hong Kong reported that an increase in bacterial density of *S. suis* in raw pork meats in wet markets occurs in hot and humid climates [23]. Collectively, the poor quality of food safety control for raw pork products at the slaughterhouses and the wet markets in this region are likely to provide the sources of this infection. The surveillance of *S. suis* contamination in pig tonsils collected at slaughterhouses and in the raw pork meats in the wet markets is being investigated in Phayao Province.

Because this study was conducted in hospitals based in Phayao Province, we cannot dismiss the possibility of incomplete coverage of the population. In addition, because the incidence rate was estimated through an observation period of over 1 year, this study should be repeated over a longer period to estimate accurately the incidence rate of this disease.

In conclusion, this population-based study demonstrated a high incidence rate of *S. suis* infection in the general population in Phayao Province, northern Thailand in 2010. Four of 31 human cases were linked epidemiologically to a local restaurant, and three cases were confirmed microbiologically as having been caused by an identical serotype 2 strain with ST1 and pulsotype A2. Combined epidemiological and molecular analyses are helpful for investigating the clustered cases of *S. suis* infection in humans. Because a large number of human cases of *S. suis* infection is estimated per year in northern Thailand, food safety control of raw pork products should be strengthened in this region.
